# Snapshot Look at Castleman Disease

**DOI:** 10.1111/jcmm.70961

**Published:** 2026-02-10

**Authors:** Ciprian Jitaru, Natalia Zlampa, Delia Dima, Anca Bojan, Mihnea Zdrenghea, Laura Urian, David Kegyes, Anamaria Bancos, Maria Santa, Andrei Ivancuta, Bobe Petrushev, Madalina Nistor, Bogdan Tigu, Catalin Constantinescu, Bogdan Fetica, Maria Puiu, Mihai‐Stefan Muresan, Ciprian Tomuleasa

**Affiliations:** ^1^ Department of Personalized Medicine and Rare Diseases, Medfuture Institute for Biomedical Research Iuliu Hatieganu University of Medicine and Pharmacy Cluj‐Napoca Romania; ^2^ Department of Hematology Iuliu Hatieganu University of Medicine and Pharmacy Cluj‐Napoca Romania; ^3^ Department of Hematology Ion Chiricuta Oncology Institute Cluj‐Napoca Romania; ^4^ Department of Oncology Bistrita Emergency Hospital Bistrita Romania; ^5^ Department of Pathology Iuliu Hatieganu University of Medicine and Pharmacy Cluj‐Napoca Romania; ^6^ The Genomics Research and Development Institute Bucharest Romania; ^7^ Department of Surgery Iuliu Hatieganu University of Medicine and Pharmacy Cluj‐Napoca Romania

**Keywords:** Castleman disease, need‐to‐know information for the clinician, snapshot look

## Abstract

Castleman disease (CD) is a rare and heterogeneous group of lymphoproliferative disorders characterised by abnormal proliferation of lymphoid tissue. First described in the 1950s, it has since been classified into two major clinical forms: unicentric CD (UCD), involving a single lymph node region and multicentric CD (MCD), which affects multiple regions and is often systemic. Further subclassification of MCD includes HHV8‐associated MCD, POEMS‐associated MCD and idiopathic MCD (iMCD), each with distinct pathophysiologic mechanisms and clinical implications. This review summarises current understanding of the epidemiology, clinical presentation, histopathology, pathogenesis and diagnostic challenges of CD. It also explores recent advances in molecular biology, including the role of interleukin‐6 (IL‐6), human herpesvirus‐8 (HHV8) and aberrant immune signalling in disease progression. Therapeutic strategies vary significantly depending on the subtype and range from surgical resection in UCD to immunotherapy, siltuximab and cytotoxic chemotherapy in MCD. Despite progress, CD remains underdiagnosed and poorly understood, especially in its idiopathic forms. Continued research into its molecular underpinnings and targeted treatments is critical to improving patient outcomes and establishing evidence‐ based guidelines.

## 1 Background on Castleman Disease

**FIGURE 1:**
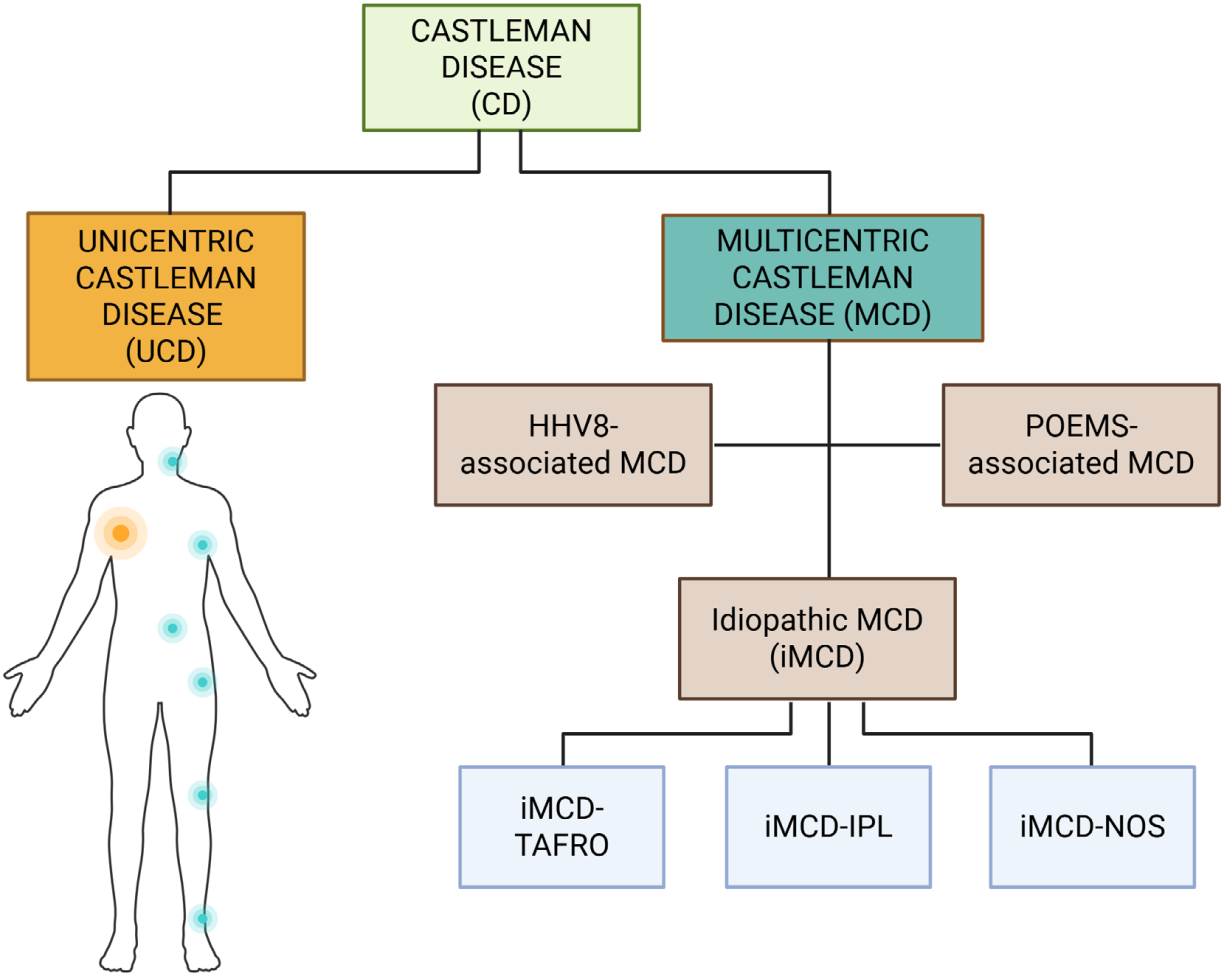
Classification of Castleman disease, according to the 5th edition of WHO classification of hematolymphoid tumours. HHV8, human herpes virus 8; IPL, idiopathic plasmacytic lymphadenopathy; NOS, not otherwise specified; POEMS, plasma cell neoplasm polyneuropathy, organomegaly, endocrinopathy, monoclonal plasma cell disorder, skin changes; TAFRO, thrombocytopenia, ascites, reticulin fibrosis, renal dysfunction organomegaly.

Lymphoproliferative disorders are a heterogeneous group of diseases characterised by the uncontrolled and exaggerated proliferation of lymphocytes (B and T cells) or their precursors, especially in immunocompromised patients, leading to monoclonal lymphocytosis, lymphadenopathy and bone marrow infiltration [1]. The main limitation of current studies lies in their small sample sizes, due to the rarity of the disease. Castleman disease (CD) is a rare condition, defined as a benign lymphoproliferative disorder of the lymph nodes. Historically, CD was classified as two main groups, based on two criteria factors: the number of swollen lymph nodes, and the systemic inflammation sympthoms [2]. MCD is further divided into three categories: KSHV/HHV8‐associated MCD (MCD HHV8+ (±HIV)), POEMS‐associated MCD and idiopathic MCD (iMCD) (Figure 1) [3]. Also, recent studies by Pierson have introduced an intermediate class of CD, named oligocentric (OCD), characterised by a limited number of 2–4 enlarged lymph nodes and systemic inflammatory symptoms at the limit between UCD and MCD; though, this subclass is not formally recognised yet [4, 5]. Though generally not considered a malignant disease, some types of CD, like UCD and MCD with two of its variants, HHV8 and iMCD, were recently listed in the classification of the 5th edition of World Health Organization Classification of Haematolymphoid Tumours: Lymphoid Neoplasms, in the category Tumour‐like lesions with B‐cell predominance [6]. CD characterisation including localisation, viral aetiology, pathogenesis, symptoms, treatments, prognosis and other details are synthesised in Table 1 [7, 8, 9, 10].

**TABLE 1:** Need‐to‐know information on the classification of Castleman disease.

Characteristics	UCD	MCD HHV8+ (±HIV)	POEMS‐associated MCD	iMCD (HHV8−)
Localisation	A single lymph node or region is affected	Multiple lymph nodes are affected	Multiple lymph nodes are affected	Multiple lymph nodes are affected
Viral aetiology	—	HHV8 positive (frequently associated with HIV infection)	HHV8 negative	HHV8 negative
Pathogenesis	Clonal expansion of stromal cells in lymph nodes	vIL‐6 production by HHV8 in infected B cells	Dysregulated cytokine secretion (IL‐6 hyperproduction)	Dysregulated cytokine secretion (IL‐6 hyperproduction)
Symptoms	Rare	Frequently: fever, fatigue, cytopenias, weight loss	Frequently: POEMS‐like symptoms: polyneuropathy, organomegaly, endocrinopathy, monoclonal plasma cell disorder, skin changes	Frequently: Fever, anaemia, edema, hypoalbuminemia, organomegaly
First‐line treatment	Surgical excision of the affected lymph node	Rituximab ± ART ± etoposide in severe forms	Bone lesions +: myeloma therapy (ASCT) Bone lesions −: Siltuximab, Tocilizumab, Corticosteroids	Siltuximab (Tocilizumab as an alternative) ± corticosteroids
Adjuvant Therapy	—	Etoposide or another chemotherapeutics (e.g., R‐CHOP if refractory or Kaposi sarcoma coexists)	Bone lesions +: myeloma therapy (autologous stem cell transplant) Bone lesions −: Rituximab, Immunomodulator/ Immunosuppressant (e.g., Sirolimus, Cyclosporine), Bortezomib, Combination chemotherapy (R‐CVP, R‐CHOP. ASCT)	Rituximab + Glucocorticoids ± Immunomodulator/Immunosuppressant (e.g., Sirolimus, Cyclosporine)
Prognosis	Generally excellent after complete excision	Variable, risk of progression to lymphoma, improved with rituximab	Bone lesions +: 90% OS Bone lesions −: 27% OS	Variable, TAFRO forms have worse prognosis, most respond to Siltuximab
Special forms	—	Kaposi sarcoma or HHV8+ lymphoma	—	iMCD‐NOS, iMCD‐TAFRO; treatment depends on subtype, iMCD‐ILP

Abbreviation: ASCT, autologous stem cell transplant.

HHV8‐associated MCD was diagnosed more commonly in immunosuppressed patients, especially patients already diagnosed with HIV (CD4+ impaired), but also, more rarely, in individuals following immunosuppressive therapy for transplant, and also in patients living with congenital haematological immunodeficiencies. These groups of patients are the most susceptible due to the defective immunosurveillance that plays the great substrate for uncontrolled HHV8 replication and the according cytokine storm onset [2, 11, 12, 13].

According to the Global Autoimmune Institute, the most prevalent age to develop CDS is highly linked to the clinical subtype. In brief, UCD is mostly reported in the young adult population, around 30–40 years old, while MCD, especially the HHV8 positive subtype, has a later onset, between 50 and 60 years old, with earlier debut in HIV‐positive cases or associated with different forms of cancer [14]. Additionally, a study from 2014 conducted by Robinson et al., identified that Caucasians have a higher risk of developing CDS, and males are more liable than females, making CDS especially prevalent in western societies [15].

This rare condition was first described by Dr. Benjamin Castleman in 1954 in a 40‐year‐old patient presenting with fever, weakness and a large mediastinal mass observed on chest X‐ray, initially suspected to be a thymoma. Subsequently, he and his colleagues documented additional similar cases, solidifying the recognition of this clinic‐pathological entity.

Initially considered a single entity, MCD was later differentiated and in 1995, the association between HHV8 and MCD was established, especially in HIV‐positive patients [16]. UCD involves a single lymph node or region, often asymptomatic. CD is a challenging pathology in terms of diagnostic complexity, pathogenesis dilemma, high heterogeneity and low incidence. Its complexity also resides in the three, overlapping subtypes of characterisation: etiological, pathological and clinical. Historically, the CD was classified according to its clinical features: unicentric (UCD) and multicentric (MCD).

### 1.1 UCD

UCD is defined as a single enlarged lymph node or lymph node region, often with mild or no known systemic symptoms. UCDs are commonly hyaline vascular and show regressed germinal centers, hyperplastic follicular dendritic cells (FDS) and reactive increased vascularity. In UCD, IL‐6 is not considered a disease driver and surgical excision is typically curative, with no major systemic manifestation [2, 17, 18].

### 1.2 MCD

MCD includes multiple lymph node regions with systematic inflammation. MCD is furtherly divided into three subsequent categories:

#### 1.2.1 HHV8‐Associated MCD

HHV8‐associated MCD typically manifests in individuals with compromised immune systems, such as those who are HIV‐positive. From a pathological perspective, patients exhibit either plasmacytic or mixed characteristics. The underlying cause of the disease is the infection of B cells by HHV8, which results in the synthesis of a viral analogue of IL‐6. This process triggers a cytokine storm, leading to systemic symptoms including fever, night sweats and organomegaly [2, 17, 19].

#### 1.2.2 POEMS‐Associated MCD

POEMS‐associated MCD occurs within the framework of POEMS syndrome (which encompasses polyneuropathy, organomegaly, endocrinopathy, monoclonal plasma cell disorder and skin alterations). The pathology frequently exhibits plasmacytic characteristics. The fundamental effects involve a monoclonal plasma cell disorder (characterised by elevated levels of VEGF and cytokines, particularly IL‐6). Nevertheless, its pathogenesis diverges from that of other MCD types [2, 17].

#### 1.2.3 Idiopathic MCD (iMCD)

Idiopathic MCD (iMCD) is characterised by being HHV8 negative and having an unknown cause. The pathology associated with this condition varies, often showing a predominance of plasmacytic or mixed features. Although human IL‐6 is responsible for this subtype of the disease in numerous instances, there are patients who exhibit normal IL‐6 levels, indicating the potential involvement of other cytokines [3, 20]. The iMCD is further categorised as follows:

##### 1.2.3.1 iMCD‐TAFRO

This condition is marked by thrombocytopenia, anasarca, fever, renal dysfunction/reticulin fibrosis and organomegaly. There exists a subset that presents with a rapid onset, a severe inflammatory phenotype and small volume lymphadenopathy. While elevated IL‐6 levels are frequently observed, they are not universally present [3, 20].

##### 1.2.3.2 iMCD‐IPL

Defined by idiopathic plasmacytic lymphadenopathy, either subacute or chronic lymphadenopathy, anaemia and polyclonal hypergammaglobulinemia. The pathology is marked by significant plasmacytosis [3, 20].

##### 1.2.3.3 iMCD‐NOS

This subtype combines the cases that do not meet criteria for TAFRO, or IPL. It may mimic an indolent lymphoma or an autoimmune disorder [3, 20].

To sum up, UCD is characterised by its localised nature and is not primarily influenced by IL‐6, while the different forms of MCD are multicentric and possess a systemic inflammatory aspect that is mainly driven by cytokines, including IL‐6, among others. The causes, clinical manifestations and pathological characteristics of the various subtypes of iMCD further demonstrate their heterogeneity and individuality [21].

The histopathological classification divides CD into three patterns: (1) hyaline‐vascular, (2) plasma cell and (3) mixed histopathological subtypes [17]. The first one, the hyaline‐vascular pattern, is mostly seen in UCD, and is characterised by specific histological aspects like the onion‐skinning concentric mantle zones, high vascular proliferation, with hyalinised vessels that cross the germinal centers, known as ‘lollipop’ lesions, small or atrophic germinal centers and dysplastic follicular dendritic cells (FDC). This CD variant can be a precursor of follicular dendritic cell sarcoma (FDCS) [17, 22, 23, 24].

The second subtype the plasma cell rich variant also occurs most often in MCD, particularly in iMCD‐IPL and POEMS‐associated MCD, and is described histologically by infiltrating sheets of mature plasma cells in the inter‐follicular areas, hyperplastic germinal centers and less evident vascularity. Clinically, the plasma cell rich variant is associated with polyclonal hypergammaglobulinemia, anaemia, and a more indolent course (usually with a good response to anti‐IL‐6 therapy) [18, 25]. Each subtype has different clinical and histopathological features and may respond differently to anti‐IL‐6 therapy [2, 26].

Finally, cases of MCD with features of both hypervascularity and plasmacytosis (mixed histology or ‘intermediate’ histology) do arise, especially in iMCD‐NOS. Such cases should be considered with combinations of clinical and laboratory parameters as histopathology alone does not yield sufficient information for accurate subtyping. As supported by machine learning‐based histopathological criteria, these patterns exhibit features of both TAFRO and IPL subtypes. Therefore, for an accurate diagnosis, the dominant pathological and clinical characteristics should be considered for approaching the most effective therapy [27]. The aetiology of CD still requires more knowledge for a better understanding, but there have been described some factors that lead to its onset, like defective immunoregulation, which causes B lymphocytes and plasma cells to accumulate abundantly in the lymphoid organs. This can be the result of multiple conditions, such as angiogenesis, chronic low‐grade inflammation, dysregulation of cytokine modulation, lymphoid‐hamartomatous hyperplasia, viral infection and, immunodeficiency linked with HHV8 infection, the main cause of CD‐20 and IL‐6 deregulation [28, 29, 30].

MCD involves multiple lymph nodes, with systemic symptoms. MCD is further classified into two major etiological categories: HHV8‐associated MCD, with or without HIV infection and more frequent in immunocompromised patients; and idiopathic MCD, HHV8 negative, without a known infectious trigger.

**FIGURE 2:**
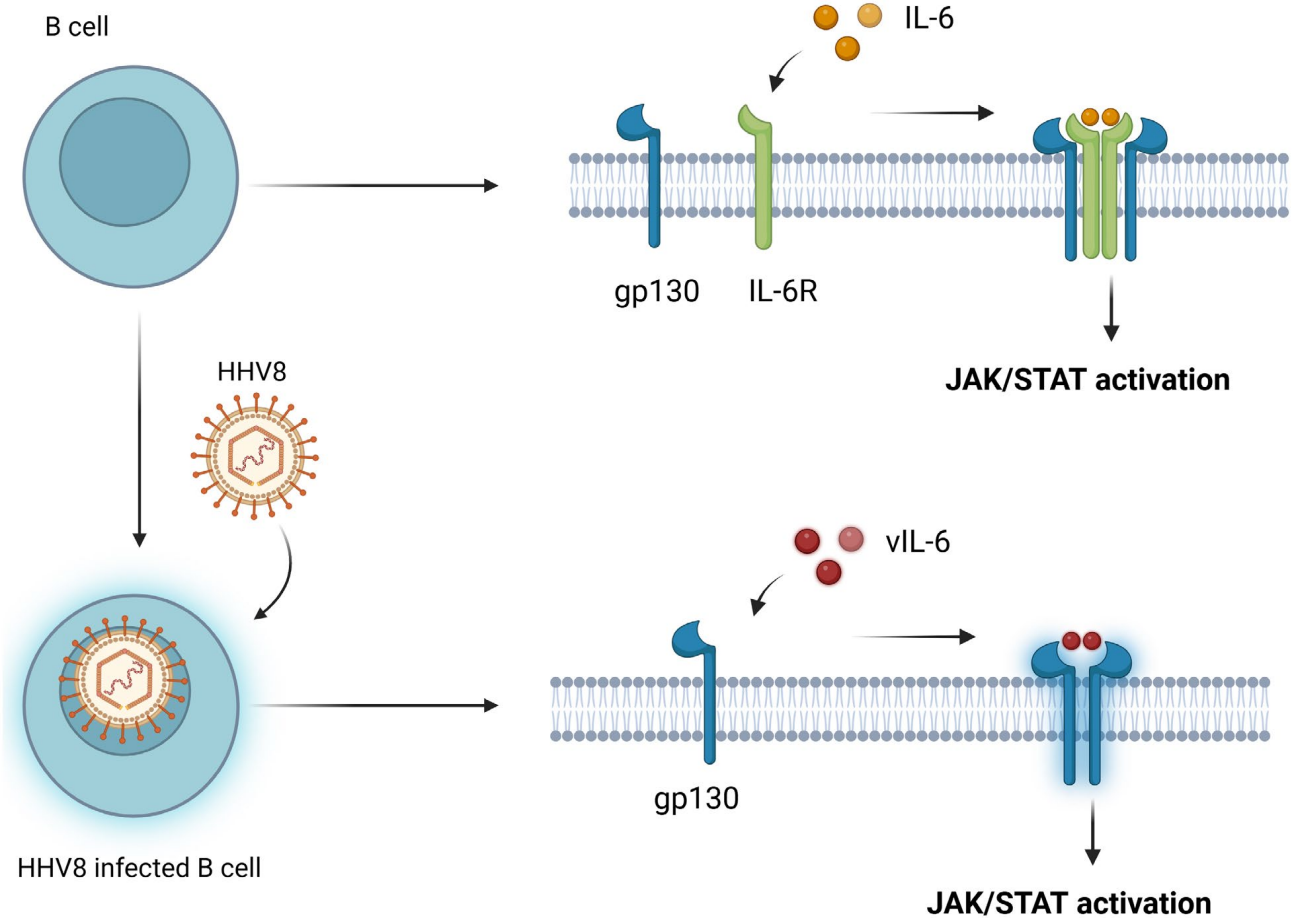
IL‐6 signalling pathway in normal versus HHV8 infected cells. In the case of HHV8+ MCD, the virus targets B cells and produces its viral IL‐6 (vIL‐6) that acts like human IL‐6 and causes inflammation, being able to bind directly to the signal transducer gp130, without the necessity of IL‐6R.

The etiopathogenesis of CD differs significantly between the unicentric and multicentric forms. In UCD, the pathogenesis is believed to involve localised clonal expansion of follicular dendritic cells and stromal elements, possibly representing a benign neoplastic process. UCD is typically not associated with systemic inflammation or elevated cytokine levels. In contrast, MCD, particularly iMCD, is characterised by a systemic inflammatory syndrome driven by elevated cytokine levels, most notably IL‐6. This hypercytokinemia is responsible for many of the systemic manifestations seen in MCD, including fever, fatigue, anaemia, hypoalbuminemia and organomegaly. In HHV8‐positive MCD, the virus infects B cells and produces a viral homologue of IL‐6 (vIL‐6), which mimics the activity of human IL‐6 and contributes to the inflammatory state. Co‐infection with HIV further amplifies this process due to immune dysregulation (Figure 2) [31].

In iMCD, no viral trigger is present and the source of excess IL‐6 production remains unclear. The activation of the JAK/STAT3 signalling pathway and overexpression of vascular endothelial growth factor (VEGF) also play important roles in pathogenesis. Some authors propose that autoimmunity or somatic mutations in haematopoietic or stromal cells could contribute to the cytokine storm characteristic of iMCD [31, 32]. Further research is nevertheless needed to fully elucidate the molecular and immunological mechanisms underlying this disease.

## 2 Clinical Symptoms and Pathophysiology

The clinical presentation of CD varies depending on the subtype and distribution of the disease. UCD is frequently asymptomatic and is often discovered incidentally during imaging or surgical procedures performed for unrelated reasons. In some cases, clinical manifestations may arise due to the mass effect of an enlarged lymph node, depending on its anatomical location; common symptoms include cough, dyspnea or dysphagia when the mass compresses adjacent structures. Unlike the multicentric form, UCD is typically not associated with systemic inflammation and inflammatory markers usually remain within normal limits [3, 21, 33].

The pathophysiology of CD is characterised by abnormal lymphoproliferation along with dysregulated cytokine production, most markedly IL‐6. In UCD, localised activation of stromal cells and an increase in angiogenesis promote the enlargement of lymph nodes with minimal systemic cytokine. In contrast, MCD is characterised by overproduction of cytokines coming from either HHV8‐infected plasmablastic cells in HHV8‐related MCD or from host immune cells in iMCD, which leads to systemic inflammation and abnormalities of many organ systems. In the case of POEMS‐related MCD, the monoclonal plasma cell disease leads to excess production of IL‐6 and VEGF, resulting in CD‐like changes in lymph nodes and multisystem involvement. The cytokine storm in MCD leads to constitutional symptoms, cytopenias and organ dysfunction in these patients [2, 17, 18, 34]. These insights will be further discussed in more detail below.

### 2.1 The Role of HHV8 in Producing Viral IL‐6

HHV8 infection stimulates the synthesis of viral (vIL‐6) in individuals with HHV8‐positive MCD by targeting B cells, especially plasmablasts, which subsequently produce vIL‐6 during the lytic phase of viral replication. vIL‐6 serves as a functional counterpart to human IL‐6, distinguished by its ability to directly bind and activate the gp130 signal transducer independently of the IL‐6 receptor. This specific property allows vIL‐6 to initiate JAK/STAT signalling across a wider array of cell types compared to human IL‐6. Predominantly found in the endoplasmic reticulum, vIL‐6 can signal within cells, promoting survival, proliferation and resistance to apoptosis, while also facilitating viral replication and latency [35, 36, 37].

In the context of HHV8‐positive MCD, vIL‐6 works along with human IL‐6 to induce the cytokine storm, resulting in systemic inflammation, fever, anaemia, hypoalbuminemia and multiorgan dysfunction [12, 38, 39]. Both vIL‐6 and human IL‐6 levels rise during disease exacerbations, and their synergistic effects are linked with more severe clinical manifestations, such as elevated C‐reactive protein, hyponatremia and increased viral load. Additionally, vIL‐6 induces the plasmacytic differentiation of B cells, which contributes to the polyclonal proliferation of plasmablasts and the lymphadenopathy observed in MCD [38, 39]. Consequently, the pathogenesis and clinical features of HHV8‐positive MCD are intrinsically associated with the excessive production and biological function of vIL‐6, which enhances inflammatory and proliferative signalling within the affected tissues [12, 38].

### 2.2 The Cytokine Storm Involving Human IL‐6, VEGF and Other Proinflammatory Mediators

The cytokine storm characterised by human (IL‐6, vIL‐6, VEGF) and various other proinflammatory mediators plays a crucial role in the pathogenesis and clinical features of MCD particularly in cases that are positive for HHV8. In instances of HHV8‐positive MCD, the virus infects B cells, especially plasmablasts, which subsequently produce vIL‐6 during the lytic phase of replication. Unlike human IL‐6, vIL‐6 can activate gp130 signalling without the need for the IL‐6 receptor, resulting in extensive activation of the JAK/STAT pathway, which promotes B‐cell proliferation, plasmacytic differentiation and resistance to apoptosis. Additionally, human IL‐6 is secreted by both infected and uninfected cells, further intensifying the inflammatory response [32, 38, 39].

The synergistic effects of vIL‐6 and human IL‐6 lead to a cytokine storm that manifests as systemic inflammation, fever, anaemia, hypoalbuminemia and multiorgan dysfunction. This process is accompanied by increased levels of VEGF, produced by stromal and follicular dendritic cells, which enhances vascular permeability, causes anasarca and leads to organomegaly. Furthermore, other cytokines such as IL‐10, TNF‐α and IL‐1β are also found at elevated levels during disease exacerbations, which further aggravates the inflammatory environment [38, 40].

From a clinical perspective, the intensity of MCD flares is directly related to the levels of both vIL‐6 and human IL‐6, with elevated concentrations correlating with more severe symptoms, increased C‐reactive protein levels and more pronounced laboratory abnormalities such as hyponatremia [38]. Therefore, the cytokine storm serves as the primary mechanism driving the distinctive systemic manifestations and organ dysfunction observed in HHV8‐positive MCD [12, 38].

The established links between MCD—notably HHV8‐positive MCD, which is marked by a cytokine storm involving human IL‐6, vIL‐66, vascular endothelial growth factor and various proinflammatory mediators—and autoimmune or autoinflammatory conditions, including the detection of autoantibodies during laboratory assessments, hold significant clinical implications for multiple reasons.

Firstly, the clinical and laboratory characteristics of MCD, particularly idiopathic MCD (iMCD), frequently overlap with those of systemic autoimmune and connective tissue disorders, such as systemic lupus erythematosus, Sjögren's syndrome and myositis. This overlap encompasses symptoms (fever, lymphadenopathy, cytopenias, organomegaly), laboratory results (elevated inflammatory markers, polyclonal hypergammaglobulinemia and autoantibody positivity) and histopathological characteristics, complicating accurate diagnosis and necessitating meticulous exclusion of autoimmune similarities [3, 19, 41, 42].

Secondly, recent research has indicated that autoantibodies—including those targeting common connective tissue disease antigens and anti‐cytokine autoantibodies—are more prevalent in iMCD patients compared to healthy individuals. This finding implies that autoimmunity or disrupted immune tolerance may play a role in the pathogenesis of iMCD, and that the cytokine storm may act as both a catalyst and a result of immune dysregulation. In the case of HHV8‐positive MCD, although the primary instigator is viral infection and vIL‐6 production, the consequent immune activation can also result in autoantibody generation and manifestations that resemble autoimmune diseases [19, 38, 41].

Lastly, the detection of autoantibodies in MCD does not inherently signify a primary autoimmune disorder but rather reflects the significant immune activation and B‐cell/plasma cell hyperactivity that is characteristic of the disease. This highlights the necessity of integrating clinical, laboratory and histopathological information to differentiate MCD from primary autoimmune or autoinflammatory diseases.

The pathogenesis of MCD linked to POEMS syndrome is primarily dependent on a monoclonal disorder of plasma cells, causing hyperproduction of cytokines, particularly VEGF and IL‐6. The monoclonal population of plasma cell origin, with predominance in lambda chain restricted conformation, is fundamentally important in the pathogenesis of the disease and is responsible for the diverse manifestations of POEMS syndrome like polyneuropathy, organomegaly, endocrinopathy, skin changes and sclerotic bone lesions [43, 44]. Regarding the histopathology of lymph nodes, this subtype of MCD is characterised by reduced germinal centers and marked polyclonal and monoclonal infiltration of plasma cells. Also, the excessive production of VEGF and IL‐6 is responsible in part for the increased vascular permeability, volume overload and systemic inflammatory response. Additionally, the high levels of IL‐6 serve as an important mediator of the lymphoproliferative changes and the constitutional symptoms [13, 44]. POEMS‐associated MCD differs from iMCD, which has no associated monoclonal plasma cell dyscrasia, but is essentially a paraneoplastic syndrome. Also, the underlying pathogenesis is usually not associated with any HHV8 infection, although certain rare instances of overlap may occur [45, 46]. The excessive cytokine production is what differentiates the POEMS‐associated MCD from other plasma cell dyscrasias and underlies its clinical and pathological differences [8, 44].

Multicentric CD typically presents with prominent systemic symptoms and laboratory abnormalities due to widespread inflammation. Common clinical features include persistent fever, profound fatigue, unexplained weight loss, generalised lymphadenopathy, hepatosplenomegaly, peripheral edema, ascites, pleural or pericardial effusions (due to hypoalbuminemia), skin changes (e.g., hemangiomas in POEMS syndrome), neuropathy (in POEMS or TAFRO subtypes). Paraclinical findings often include elevated inflammatory markers, anemia (usually normocytic, normochromic), thrombocytopenia (especially in TAFRO syndrome), hypoalbuminemia, hypergammaglobulinemia (polyclonal), elevated IL‐6 levels (not routinely available), increased VEGF (in POEMS), negative tests for HHV8 and HIV (in idiopathic forms) [20, 47, 48, 49]. Imaging studies, particularly contrast‐enhanced computed tomography (CT) and positron emission tomography (PET‐CT), play an essential role in the evaluation of CD. Findings typically reveal diffuse, mildly hypermetabolic lymphadenopathy involving multiple nodal regions. Additionally, organomegaly, most commonly hepatomegaly and splenomegaly, is frequently observed. Importantly, imaging usually does not demonstrate focal neoplastic lesions, which helps distinguish CD from malignant lymphomas or solid tumours.

Histopathological examination remains the cornerstone for diagnosis and allows differentiation between histological variants Early diagnosis is essential due to the risk of progression to severe systemic disease, particularly in multicentric forms [8, 50]. CD can affect individuals of any age or gender, although it is most commonly diagnosed in middle‐aged adults. In general, no well‐established risk factors have been identified for the idiopathic forms of the disease. However, patients infected with HHV8, particularly those who are also HIV‐positive, have a significantly increased risk of developing HHV8‐associated MCD. In these cases, viral infection and immunosuppression appear to play a central role in disease pathogenesis [51]. Treatment of HHV8 positive MCD: In the context of HHV8 positive MCD, unlike iMCD, Siltuximab is not an efficient therapeutic approach due to the presence of the viral IL‐6, which cannot be neutralised by this agent. For this subtype, Rituximab, an anti‐CD20 monoclonal antibody, is the first‐line therapy recommended, which can be combined with chemotherapeutics like etoposide or doxorubicin, in more aggressive pathologies or with antiretroviral agents, like ganciclovir or valganciclovir, in HIV‐positive patients. Its main target is to deplete the B cells that are affected and produce overexpression of IL‐6 [2, 52, 53].

In the case of unicentric CD, complications are exceedingly rare, particularly given the excellent prognosis following complete surgical excision. However, a very low risk exists for the development of a potentially life‐threatening autoimmune condition known as paraneoplastic pemphigus. For this reason, patients with UCD are monitored for signs of autoimmune involvement, especially in the months following treatment. Paraneoplastic pemphigus is a rare autoimmune blistering disorder that affects both the skin and mucous membranes. It is considered a severe variant of pemphigus and is most often associated with underlying malignancies or lymphoproliferative disorders, including UCD in some cases. The disease is characterised by painful vesiculobullous lesions and carries a poor prognosis, particularly when pulmonary complications such as bronchiolitis obliterans are present.

In multicentric CD, complications primarily result from the excessive secretion of IL‐6, which promotes chronic inflammation and immunodeficiency. This dysregulation of the cytokine cascade contributes to hematologic complications, such as inflammatory responses and thromboembolic events, as well as to multi‐organ involvement, particularly affecting renal and hepatic function. Moreover, an increased risk of malignancies has been observed among patients with idiopathic MCD (iMCD), underscoring the importance of long‐term monitoring and oncologic surveillance in this population [50, 51, 54, 55].

## 3 Diagnosis and Staging

Unicentric CD presents according to the pathology diagnosis in two main variants: the hyaline‐vascular type, which is the most common, and the plasmocytic type, which is rare and accounts for approximately 10% of cases. The hyaline‐vascular variant is characterised by regressed or atrophic germinal centers, which are often hyalinised and contain very few lymphocytes. Typically, two adjacent germinal centers are surrounded by a prominent mantle zone. The mantle zone displays a concentric arrangement of lymphocytes, creating an ‘onion‐skin’ appearance under microscopic examination. Follicles are atrophic and hyalinised, with a marked reduction in lymphoid content. A distinctive feature is the proliferation of blood vessels that penetrate the germinal centers in a radial pattern, giving rise to a ‘lollipop’ appearance. In addition, there is prominent vascular proliferation throughout the lymph node, while the lymphatic sinuses are usually inconspicuous or absent. The plasmocytic variant is defined by a polymorphous interfollicular plasma cell infiltrate. The interfollicular lymphoid tissue contains numerous small blood vessels, contributing to the hypervascular appearance of the node. The reactive germinal centers are typically enlarged and hyperplastic, while the lymph node architecture is generally preserved. Unlike the hyaline‐vascular type, the lymphatic sinuses remain open and unobstructed, without significant architectural distortion [56, 57, 58].

A key factor influencing prognosis and therapeutic response in CD is the histopathological subtype. In the multicentric form, the involvement of multiple lymph node regions is associated with systemic manifestations and more complex clinical outcomes. MCD is histologically classified into three main variants: hypervascular, plasmocytic and mixed. The hypervascular variant shares many histological features with the hyaline‐vascular subtype seen in UCD. It typically lacks dysplastic follicular dendritic cells and is most frequently observed in patients with TAFRO syndrome. The lymph nodes show regressed germinal centers, concentric layering of mantle zone lymphocytes and increased vascular proliferation; however, follicular dendritic cell abnormalities are absent. The plasmocytic variant is characterised by a dense infiltrate of mature plasma cells in the interfollicular areas. Germinal centers tend to be atrophic or regressed but generally maintain their structural integrity. Compared to the hypervascular type, this variant exhibits less prominent vascularisation. It is frequently associated with hypergammaglobulinemia and systemic inflammatory features, including elevated IL‐6 levels [20, 56].

In UCD, imaging commonly shows a single enlarged lymph node, most often located in the cervical, mediastinal or abdominal regions. MCD is characterised by multiple lymphadenopathies, often associated with splenomegaly and ascites. iMCD typically presents with symmetric, generalised lymphadenopathy and splenomegaly. HHV8 testing via immunohistochemistry or PCR is not necessary for UCD but is essential for confirming MCD. HHV8 positivity in lymph node or blood is a hallmark of HHV8‐associated MCD, while iMCD is negative for HHV8. Similarly, HIV testing is usually positive in HHV8‐associated cases and negative in iMCD and UCD.

Exclusion of other causes is important, particularly in atypical presentations. In MCD, lymphomas associated with HHV8 or EBV and HIV‐related malignancies must be ruled out. In iMCD, autoimmune disorders, infections and lymphomas should be excluded through appropriate investigations. Laboratory tests in UCD are usually normal or only mildly altered. In contrast, MCD often shows anaemia, elevated inflammatory markers and hypoalbuminemia. In iMCD, laboratory abnormalities are evaluated according to established international diagnostic criteria.

For UCD, diagnosis is confirmed by identifying a hyperplastic solitary lymph node through biopsy. In MCD, diagnosis is based on histological findings and HHV8 positivity in conjunction with the clinical picture. iMCD is diagnosed based on established international criteria, which require the presence of both major criteria, characteristic histopathological findings and generalised lymphadenopathy, in addition to at least two minor criteria, one of which must be a laboratory abnormality. Furthermore, diagnosis mandates the exclusion of other conditions that may mimic iMCD, including infectious, autoimmune and malignant disorders [2, 20, 56].

The differential diagnosis of CD is both essential and complex, as it must distinguish this rare lymphoproliferative disorder from a wide range of other conditions. These include hematologic malignancies such as Hodgkin lymphoma and non‐Hodgkin lymphomas, as well as autoimmune diseases like systemic lupus erythematosus. Solid tumours must also be excluded, particularly when lymphadenopathy is the initial or dominant clinical feature. Other considerations include primary amyloidosis, Kaposi sarcoma, follicular dendritic cell tumours and angioimmunoblastic T‐cell lymphoma (AITL). Accurate diagnosis requires a combination of clinical, histopathological, immunophenotypic and imaging assessments to differentiate CD from these entities with overlapping features [55, 59].

The prognosis of CD varies significantly depending on the subtype. In UCD, the outlook is excellent, especially following complete surgical resection, with a reported 10‐year survival rate exceeding 95%. In cases where resection is not feasible or incomplete, radiotherapy may be employed, although the 20‐month survival rate decreases to approximately 82%. For patients with HHV8‐associated MCD, who respond well to treatment with rituximab, the 2‐year survival rate can exceed 94%. However, in patients who fail to respond to rituximab, treatment often escalates to chemotherapy regimens such as etoposide‐based protocols, which are associated with a less favourable prognosis.

In contrast, idiopathic MCD (iMCD) is generally associated with a poorer prognosis compared to HHV8‐positive cases, particularly in patients with TAFRO syndrome or those with a suboptimal response to anti‐IL‐6 therapy [60].

## 4 Siltuximab‐Based Therapy

**FIGURE 3:**
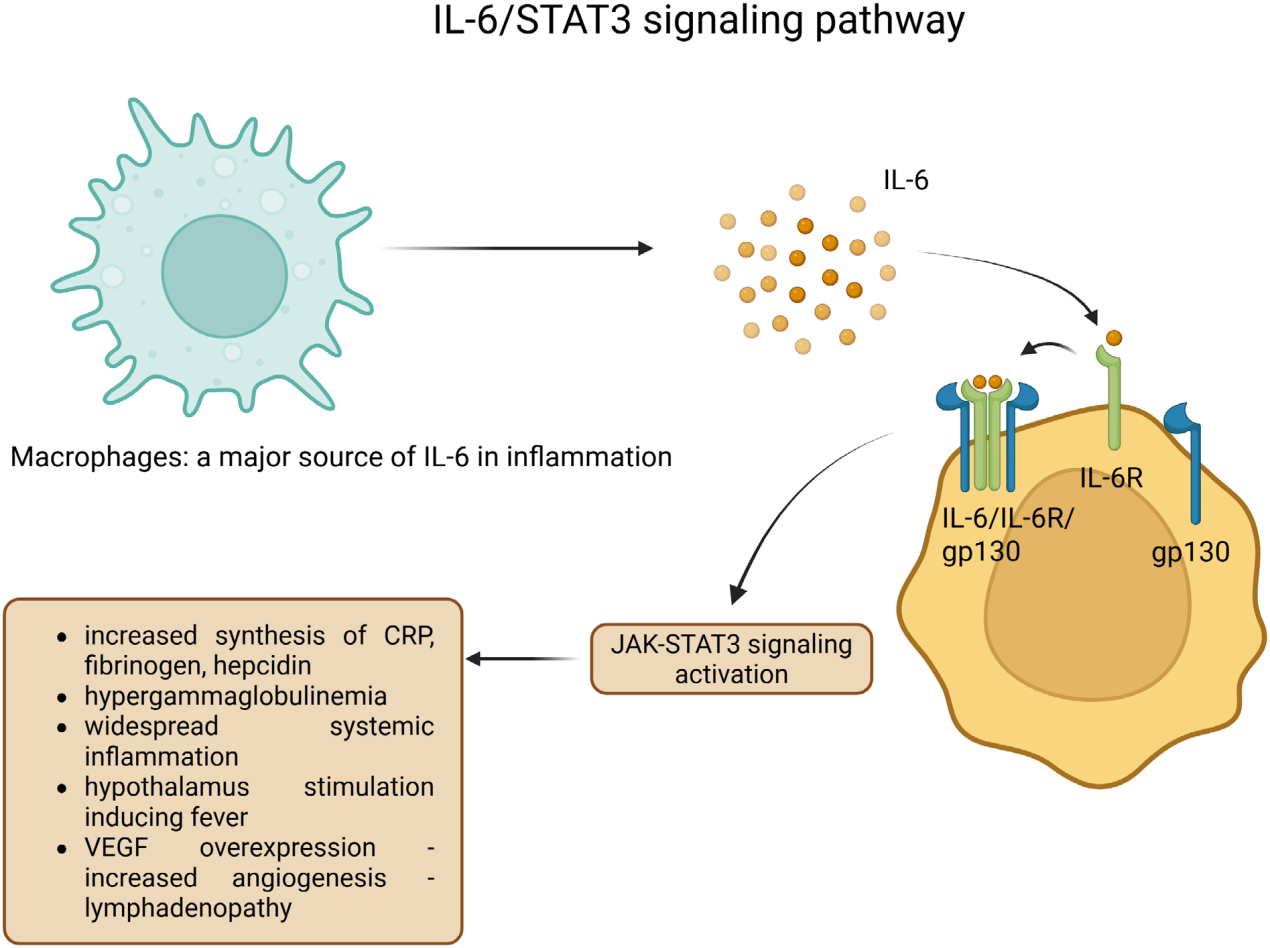
Activation of JAK/STAT3 signalling pathway through IL‐6/IL‐6R/gp130. Activated macrophages release IL‐6 molecules, which further bind to the IL‐6 receptor (IL‐6R) and then, the gp130 signal transducer dimerisation is triggered. This new conformation initiates the signalling pathway JAK/STAT3, resulting in systemic inflammatory responses such as acute‐phase protein synthesis, fever onset and angiogenesis.

Siltuximab is a monoclonal antibody that targets IL‐6. By binding to IL‐6, it prevents IL‐6 from interacting with its receptor (IL‐6R), thereby attenuating the proinflammatory effects of this cytokine. In the classical signalling pathway, IL‐6 binds to its membrane‐bound receptor (IL‐6R), which is expressed on T cells, hepatocytes and neutrophils. This IL‐6–IL‐6R complex associates with the gp130 co‐receptor, triggering the intracellular JAK/STAT3 signalling cascade. Activation of this pathway leads to a cascade of systemic effects (Figure 3), including increased synthesis of acute‐phase reactants such as C‐reactive protein (CRP), fibrinogen and hepcidin. It also triggers B‐cell activation, resulting in hypergammaglobulinemia and T‐cell activation, which contributes to widespread systemic inflammation. Additionally, the pathway stimulates the hypothalamus, leading to fever and promotes overexpression of VEGF, which enhances angiogenesis and contributes to the development of lymphadenopathy. This will lead in CD patients to lymph node hyperplasia, systemic inflammatory syndrome (fever, elevated CRP, anemia, fatigue), bone marrow plasmacytosis, occasionally, VEGF‐mediated syndrome (edema, ascites) [61].

**FIGURE 4:**
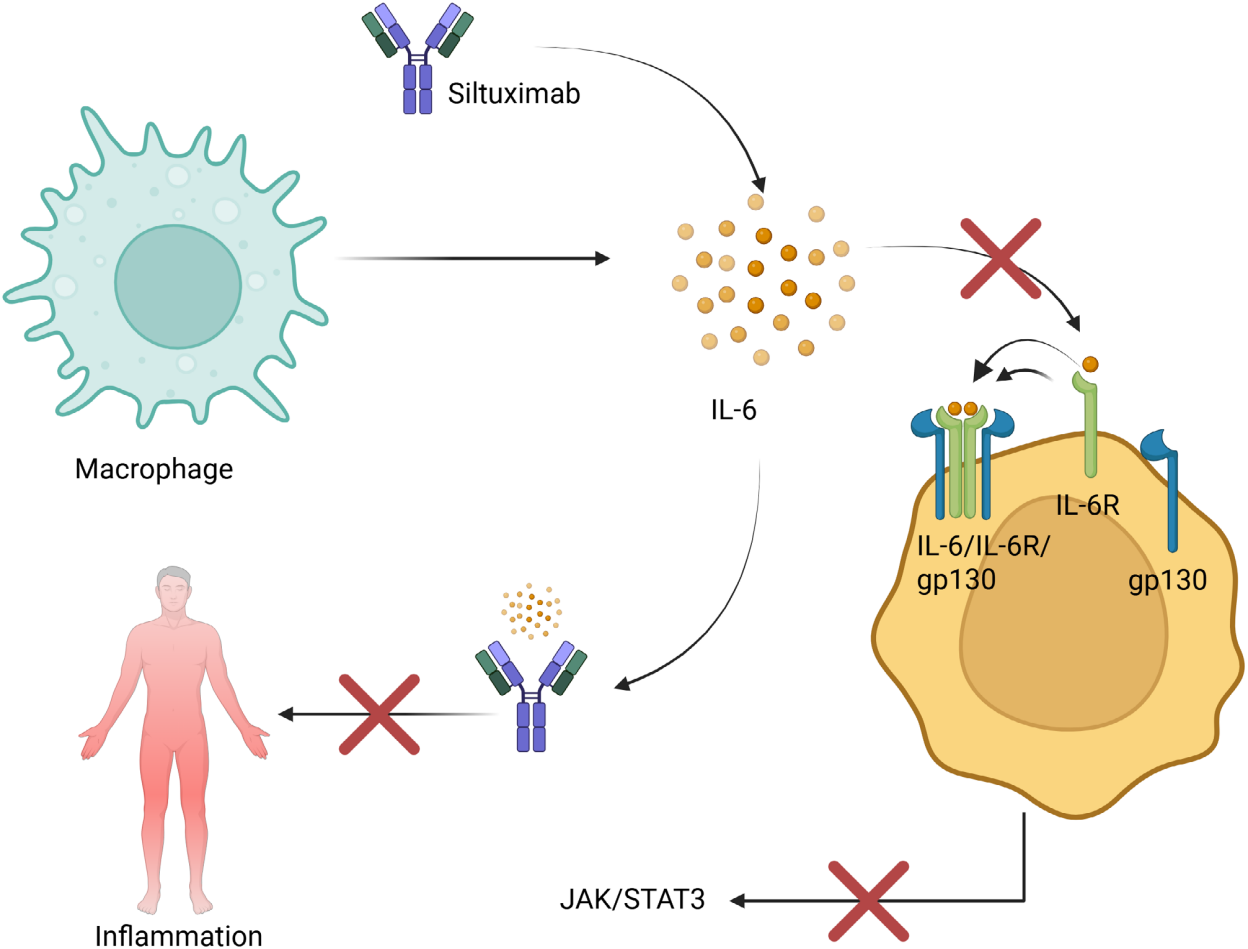
Siltuximab‐mediated inhibition of IL‐6 signalling in inflammation. Siltuximab is a monoclonal antibody that binds to IL‐6, which blocks its capacity to interact with its receptor, IL‐6R and the subsequent dimerisation of gp130 and the ultimate formation of the IL‐6/IL‐6R/gp130 complex. Consequently, the JAK/STAT3 pathway remains inactive and the resulting effect of Siltuximab is the control and reduction of the overall inflammation in the organism.

Siltuximab exerts its therapeutic effect by binding directly to IL‐6, thereby preventing its interaction with the IL‐6 receptor (IL‐6R) and the gp130 co‐receptor (Figure 4). This inhibition effectively blocks activation of the JAK/STAT3 signalling pathway, which is central to the pathophysiology of iMCD.

As a result, the production of acute‐phase proteins such as C‐reactive protein (CRP), fibrinogen and hepcidin is reduced, leading to decreased systemic inflammation. Clinically, this manifests as resolution of fever, improvement in constitutional symptoms such as fatigue and anaemia and a reduction in lymph node size [62, 63, 64]. Siltuximab has been approved by both the FDA and EMA under the brand name Sylvant for intravenous use in patients with HIV‐negative and HHV8‐negative MCD. Its introduction in 2016 marked a significant advancement in managing this rare disease [65], also confirmed in the real‐life setting [8].

## 5 Alternative Therapies Following Siltuximab Relapse

Following siltuximab relapse, when CD, particularly idiopathic iMCD, relapses following treatment with siltuximab, therapeutic strategies must be reassessed and tailored to disease severity, patient comorbidities and response to prior interventions [66]. Siltuximab is an anti–IL‐6 monoclonal antibody and the standard first‐line therapy for iMCD, but not all patients respond fully or maintain long‐term remission [64]. In relapsed or refractory cases, several second‐line treatment options are available. In patients who relapse after siltuximab, switching to tocilizumab, an anti–IL‐6 receptor monoclonal antibody, may be considered. Although data are limited, some patients who do not respond to siltuximab may still derive benefit from tocilizumab, potentially due to differences in their mechanisms of action. Tocilizumab can help reduce systemic symptoms and inflammatory markers, though its use requires close monitoring for infections and liver function abnormalities [53, 67]. Sirolimus, an oral mTOR inhibitor, has shown promising results in patients with siltuximab‐ refractory iMCD, particularly those with the TAFRO clinical subtype or prominent immune dysregulation [68, 69]. By targeting the mTOR pathway, which lies downstream of IL‐6 signalling, sirolimus can dampen the hyperactive immune response seen in iMCD. Drug level monitoring and prophylaxis against infections are typically required during treatment [68].

Systemic corticosteroids are frequently used for acute symptom control or as bridging therapy while awaiting the effects of longer‐acting agents such as sirolimus or tocilizumab. While steroids can provide rapid symptom relief, they are not recommended as long‐term monotherapy due to their extensive side effect profile [3, 21, 70]. For patients with aggressive, rapidly progressive or life‐threatening disease—especially when there is concern for transformation to lymphoma—cytotoxic chemotherapy regimens such as R‐CHOP or CVAD may be appropriate. These regimens are typically reserved for severe or refractory cases and are best suited for patients who can tolerate their toxicities [3, 71]. Chemotherapy may also be used as a bridge to more definitive treatments such as stem cell transplantation [72].

In cases where there is evidence of CD20‐positive B‐cell involvement, or when CD overlaps with autoimmune features, rituximab, an anti‐CD20 monoclonal antibody, may be used [71, 73]. It is more commonly employed in HHV‐8–associated MCD but can be considered in idiopathic cases with suspected autoimmune components [3]. Rituximab is often used in combination with corticosteroids or chemotherapy [53]. For select patients with relapsed, refractory or highly aggressive CD that has responded to salvage therapy, autologous stem cell transplantation (ASCT) may offer the potential for long‐term remission. Due to its intensity and associated risks, this option is generally reserved for younger, medically fit patients with chemosensitive disease [74]. Patients who do not respond to standard treatments should be referred to centers with expertise in CD to explore clinical trial options. Experimental therapies under investigation include JAK inhibitors (such as ruxolitinib), BTK inhibitors, IL‐1 inhibitors and novel mTOR pathway modulators. Participation in clinical trials may provide access to innovative therapies that address broader aspects of immune dysregulation [75].

Management of relapsed CD requires ongoing monitoring of inflammatory markers (such as CRP, fibrinogen and albumin), organ function and clinical status. Supportive care should include infection prophylaxis, nutritional support and symptom management as needed. A multidisciplinary approach, involving haematology, rheumatology, infectious disease and pathology teams, is recommended for optimal patient outcomes [2].

## 6 How I Treat CD

As a proof of principle, we will present the case of a 28‐year‐old woman who initially presented with persistent fatigue, night sweats, low‐grade fevers and significant weight loss over 3 months. On examination, she had generalised lymphadenopathy and mild hepatosplenomegaly. Laboratory studies revealed elevated CRP (95 mg/L), anemia (Hb 9.5 g/dL), hypoalbuminemia and thrombocytosis. Infectious, autoimmune and malignant workups were negative. Lymph node biopsy confirmed iMCD, plasma cell variant. HHV8 and HIV testing were negative.

For the initial management and response, the patient was started on siltuximab 11 mg/kg IV every 3 weeks, with concurrent short‐course corticosteroids for symptomatic relief. Within 8 weeks, he showed marked clinical improvement—resolution of fevers and night sweats, normalisation of CRP and improved hemoglobin value. Imaging at 12 weeks demonstrated regression of lymphadenopathy. He continued maintenance siltuximab with stable disease for approximately 18 months. Around month 20, our patient. began experiencing recurrence of symptoms including fatigue, low‐grade fevers and abdominal discomfort. Laboratory evaluation revealed rising CRP (68 mg/L), falling albumin (2.5 g/dL) and mild thrombocytopenia. Repeat imaging showed new lymphadenopathy and mild ascites. Despite re‐confirmation of diagnosis and continued siltuximab dosing, symptoms persisted and worsened over the following 2 months, suggesting loss of response to siltuximab. After multidisciplinary discussion and exclusion of lymphoma or autoimmune mimics, he was transitioned to sirolimus 2 mg daily, titrated to maintain trough levels of 5–15 ng/mL. Corticosteroids were reintroduced briefly during the transition. Within 6 weeks, the patient reported improved energy, reduction in lymph node size and normalisation of inflammatory markers. Albumin and hemoglobin levels gradually improved over the next 3 months. At his 6‐month follow‐up on sirolimus monotherapy, he remained clinically stable with no active symptoms, normalised laboratory parameters and minimal residual lymphadenopathy on imaging. The patient continues on sirolimus with regular monitoring and no significant adverse effects. He has returned to work full‐time and maintains a good performance status. This case illustrates a typical course of iMCD where an initial response to IL‐6 blockade with siltuximab is followed by relapse, but disease control is regained with targeted mTOR inhibition using sirolimus.

## 7 Conclusion

CD remains a rare and heterogeneous group of lymphoproliferative disorders that present significant diagnostic and therapeutic challenges. Advances in understanding its pathophysiology—particularly the role of IL‐6 and immune dysregulation—have led to improved classification and targeted therapies, especially for idiopathic multicentric CD. Despite these strides, there is still a critical need for standardised diagnostic criteria, better biomarkers and long‐term outcome data. Continued research and clinical collaboration are essential to optimise management strategies, improve patient outcomes and further elucidate the underlying mechanisms driving this complex disease.

## Author Contributions

All authors contributed to the design and writing of the manuscript. Ciprian Jitaru and Natalia Zlampa wrote the manuscript. Ciprian Tomuleasa supervised the work.

## Funding

The work is funded by a national grant of the Romanian Research Ministry—PNRR 1089 2024–2026 (PNRR/2022/C9/MCID/18, Contract No. 760278/26.03.2024), by an international grant of the European Commission—Horizon Europe Framework Network, HORIZON‐TMA‐MSCA‐DN (Proposal Number 101227725—‘Advancing in the CHallenge of Improving Lymphoma and Leukaemia Survival—ACHILLES’), by a bilateral collaboration grant between Romania and Moldova (PN‐IV‐P8‐8.3‐ROMD‐2023‐0036).

## Conflicts of Interest

The authors declare no conflicts of interest.

## Data Availability

Data sharing not applicable to this article as no datasets were generated or analysed during the current study.
